# [Retracted] SRF-miR-29b-MMP2 axis inhibits NSCLC invasion and metastasis

**DOI:** 10.3892/ijo.2025.5750

**Published:** 2025-05-07

**Authors:** Hong-Yan Wang, Yong-Sheng Tu, Jie Long, Hui-Qiu Zhang, Cui-Ling Qi, Xiao-Bin Xie, Shu-Hua Li, Ya-Jie Zhang

Int J Oncol 47: 641-649, 2015; DOI: 10.3892/ijo.2015.3034

Following the publication of this paper, it was drawn to the Editor's attention by a concerned reader that, in Fig. 1A and Fig. 3A and B showing the results of Transwell invasion and migration experiments, the majority of the data panels were identified as having overlapping sections, such that the affected data panels, which were intended to show the results of differently performed experiments, had all apparently been derived from the same original sources.

Owing to the large number of duplications of data that have been identified in this paper, the Editor of *International Journal of Oncology* has decided that it should be retracted from the Journal on account of a lack of confidence in the presented data. The authors were asked for an explanation to account for these concerns, but the Editorial Office did not receive a reply. The Editor apologizes to the readership for any inconvenience caused.

